# Palliative Care for Hospitalized Patients With Stroke: Results From the 2010 to 2012 National Inpatient Sample

**DOI:** 10.1161/STROKEAHA.117.016893

**Published:** 2017-08-17

**Authors:** Tarvinder Singh, Steven R. Peters, David L. Tirschwell, Claire J. Creutzfeldt

**Affiliations:** From the Department of Neurology, Harborview Medical Center, University of Washington, Seattle.

**Keywords:** end-of-life, inpatients, palliative care, stroke, subarachnoid hemorrhage, United States

## Abstract

**Background and Purpose—:**

Substantial variability exists in the use of life-prolonging treatments for patients with stroke, especially near the end of life. This study explores patterns of palliative care utilization and death in hospitalized patients with stroke across the United States.

**Methods—:**

Using the 2010 to 2012 nationwide inpatient sample databases, we included all patients discharged with stroke identified by *International Classification of Diseases-Ninth Revision* codes. Strokes were subclassified as ischemic, intracerebral, and subarachnoid hemorrhage. We compared demographics, comorbidities, procedures, and outcomes between patients with and without a palliative care encounter (PCE) as defined by the *International Classification of Diseases-Ninth Revision* code V66.7. Pearson χ^2^ test was used for categorical variables. Multivariate logistic regression was used to account for hospital, regional, payer, and medical severity factors to predict PCE use and death.

**Results—:**

Among 395 411 patients with stroke, PCE was used in 6.2% with an increasing trend over time (*P*<0.05). We found a wide range in PCE use with higher rates in patients with older age, hemorrhagic stroke types, women, and white race (all *P*<0.001). Smaller and for-profit hospitals saw lower rates. Overall, 9.2% of hospitalized patients with stroke died, and PCE was significantly associated with death. Length of stay in decedents was shorter for patients who received PCE.

**Conclusions—:**

Palliative care use is increasing nationally for patients with stroke, especially in larger hospitals. Persistent disparities in PCE use and mortality exist in regards to age, sex, race, region, and hospital characteristics. Given the variations in PCE use, especially at the end of life, the use of mortality rates as a hospital quality measure is questioned.

## Abstract 2

Supplemental Digital Content is available in the text.

Stroke is the leading cause of serious long-term disability in the United States and the fifth leading cause of death.^1,2^ The stroke illness trajectory presents a unique challenge that is distinct from most other serious illnesses;^3^ the presentation is sudden and unexpected, and whereas a small proportion of patients will receive potentially curative acute treatment, patients with severe stroke typically face treatment decisions that leave little time for deliberation and can lead either to an early death in the setting of withdrawal or withholding of life-sustaining interventions or enable survival with a wide range of disability.^3^ In some cases, survival may be worse than death.^4^ Given their neurological impairment, conversations about goals of care usually occur between providers and surrogate decision makers, rather than with the patient themselves.

These observations highlight the importance of integrating palliative care into the acute stroke care setting.^5^ Palliative care is a multidisciplinary approach to medical care that focuses on improving communication, decision making, and quality of life for patients with serious illness and their families. Early integration of palliative care into acute stroke care has been recently endorsed by the American Heart^6^/American Stroke Association^7^ and Neurocritical Care Society,^8^ but data remain limited about how to best implement these recommendations and how to measure their benefit.^9–11^ Palliative care services are available in an increasing number of US hospitals, but substantial variations remain in access and use across regional, socioeconomic, racial, and ethnic groups.^12,13^

In an effort to evaluate hospital quality of care, the Center for Medicare and Medicaid Services (CMS) currently uses 30-day mortality after ischemic stroke as a quality of care surrogate^14^ and provides access to adjusted rates on the CMS hospital compare website (https://www.medicare.gov/hospitalcompare/search.html). Inpatient mortality after stroke varies widely depending on several patient and hospital characteristics, including the hospital’s use of do not resuscitate (DNR) orders.^7,15,16^ Use of palliative care after stroke is likely also associated with higher in-hospital and 30-day mortality rates and may not indicate lower hospital quality of care: this is not accounted for in the CMS stroke mortality quality measure.

The overall goal of this study was to characterize current practices around the use of palliative care in a nationally representative sample of patients with stroke by (1) identifying patient and hospital characteristics associated with palliative care utilization, and (2) assessing how the use of palliative care influences inpatient mortality.

## Methods

### Database

We performed a retrospective observational study in patients with stroke admitted to US acute care hospitals using discharge data from the publicly available national inpatient sample (NIS), healthcare cost and utilization project, and agency for healthcare research and quality.^17–19^

The NIS is a cross-sectional, all-payer, inpatient care data set in the United States, consolidated on an annual basis. It is the largest inpatient health data set in the United States. Unweighted, it contains data from >7 million hospital stays from >1000 hospitals each year, which represent a stratified sample of 20% of all nonfederal hospitals. Weighted, it estimates >35 million hospitalizations nationally. Discharge data include demographics, socioeconomics, primary and secondary diagnoses, procedures, and length of stay (LOS). The NIS database contains deidentified information and is exempt from institution review board approval at our institution.

### Stroke Data Selection

We identified adult (age, >18 years) stroke admissions from 2010 to 2012 using *International Classification of Diseases-Ninth Revision* (ICD-9) diagnosis codes. Codes 433.X1–occlusion and stenosis of cerebral artery with infarction, 434.X1–occlusion of cerebral artery with infarction, and 436–acute but ill-defined cerebrovascular disease, irrespective of their diagnosis position, were used to identify ischemic strokes. Code 430 (first diagnosis only) was used to identify subarachnoid hemorrhage (SAH) and 431 (first diagnosis only) for intracerebral hemorrhage (ICH). Cases were excluded if there was a concomitant ICD-9 code for traumatic brain injury or rehabilitation stay.^20^

Demographic and socioeconomic factors were identified from the primary data set. Race/ethnicity had a high degree of missing data compared with other variables because of state suppression or partial reporting by hospitals. We identified individuals with intubation and PEG (percutaneous endoscopic gastrostomy) tube placement separately as proxy for life-prolonging care in these patients. In addition, cancer, heart disease, and dementia were identified because of implications for end-of-life care, and atrial fibrillation was identified because of its increased risk of large cardioembolic strokes.

#### Palliative Care

Palliative care was identified using the ICD-9-CM procedure, code V66.7 (palliative care encounter [PCE]), in the hospital discharge data. This code is added by billing staff when components of palliative care, such as comfort care, end-of-life care, and hospice care, are mentioned in the treatment record of the patient and is independent of whether or not a palliative care specialist was consulted or not.^21^ The PCE code is not used for pain and symptom management. This article uses the term PCE to indicate the presence of V66.7 code in the patient’s medical record. Several scenarios about the use of the V66.7 code in end-of-life and hospice care admissions and its interpretation by multiple national databases, such as CMS and US News and World Report, have been described.^22^ Recently, this code was examined in patients with ICH using NIS data from the previous decade.^23^

#### Death

We used the healthcare cost and utilization project database uniform discharge disposition to track death during hospitalization. We compared the timing of death in PCE versus non-PCE patients. We defined early death as death occurring with hospital LOS ≤2 days. We explored implications of early death for stroke mortality as a CMS measure of high-quality care in the setting of PCE. As the healthcare cost and utilization project database format changed in 2012, this combined analysis was limited to 2010 and 2011 data.

### Statistical Analysis

Pearson χ^2^ test was used to compare proportions between categories of PCE versus no PCE. Logistic regression was used to evaluate independent associations with PCE use. Covariates for logistic regression included age, race, sex, hospital characteristics, all-patient refined diagnosis-related group severity, and year. Statistical significance was defined as a *P* value of <0.05. Statistical analysis was performed using STATA data analysis and statistical software.

The all-patient refined diagnosis-related group, which assesses risk of mortality using an algorithm developed by 3 mol/L health information systems, was used to determine disease severity and its correlation with PCE. All-patient refined diagnosis-related group is a proprietary 4-point ordinal scale (minor, moderate, major, and extreme risk of mortality) derived from age, primary and secondary diagnoses, and procedures.^24^

## Results

We identified 395 411 adult patients with stroke. The majority of patients had ischemic strokes (86%) followed by ICH (10%) and SAH (4%). The mean age was 70.1 years (SD, 16), 52% were women and 69% were white. Among all patients with stroke, 24 641 (6.2%) received PCE, and this proportion increased with each study year from 5.4% in 2010 to 6.9% in 2012 (Table I in the online-only Data Supplement).

### Palliative Care and Patient Characteristics

Bivariate analysis of pertinent variables is presented in Table 1. Although specific stroke severity scales (National Institutes of Health Stroke Scale, ICH score, Hunt/Hess) were not available in this cohort, proxies of overall illness severity, including the all-patient refined diagnosis-related group severity subclass and codes for intubation and coma, were associated with an increased rate of PCE use, whereas PEG placement was less common among patients with PCE (Table 1).

**Table 1.**
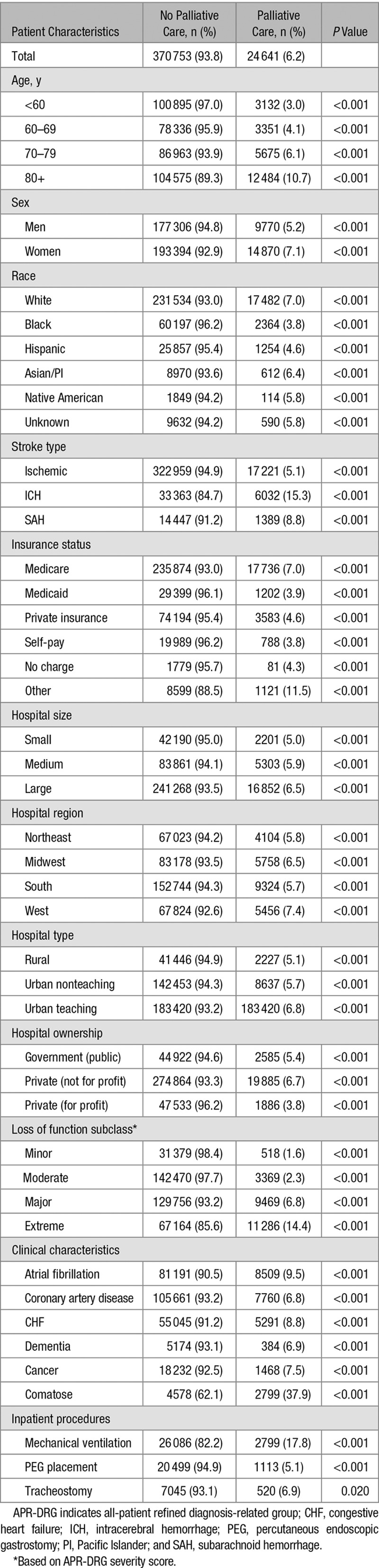
Patient and Hospital Characteristics in Relation to Palliative Care Encounter (Bivariate Analysis)

Using multivariate analysis, we found a variety of patient characteristics that were independently associated with the use of PCE (Table 2), including older age and female sex. Compared with whites, the rate of PCE use was significantly lower in blacks (odds ratio [OR], 0.62), Hispanics (OR, 0.67), and Asians (OR, 0.73). ICH, while representing only 10% of overall strokes, was associated with a higher rate of PCE use than ischemic stroke (OR, 3.40).

**Table 2.**
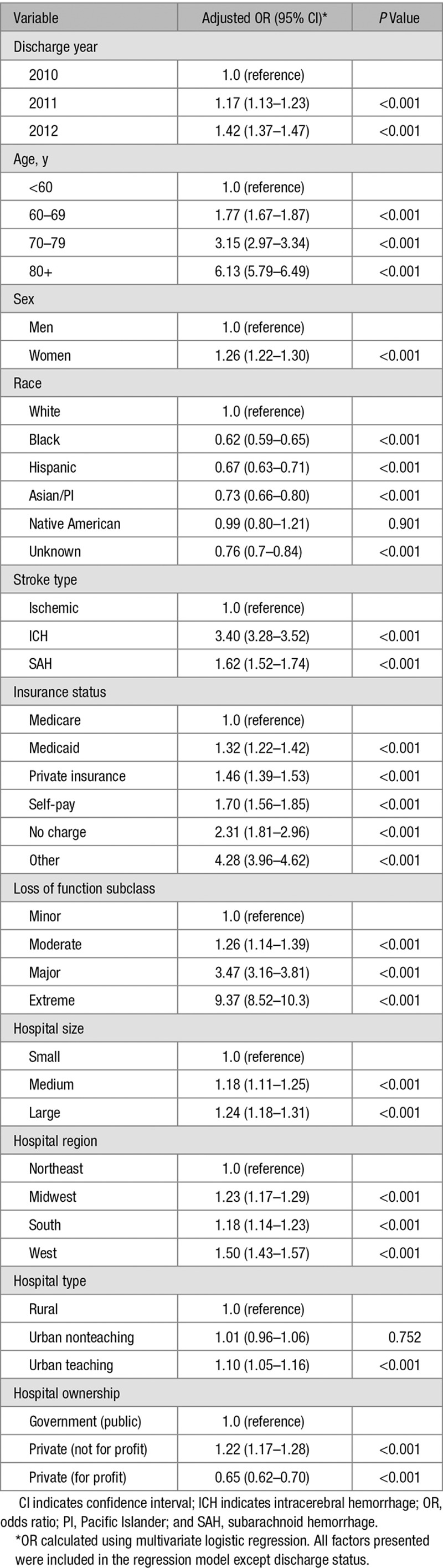
Logistic Regression: Predictors of Palliative Care Encounter

The mean LOS for all patients with stroke receiving PCE was 6.8 days (95% confidence interval, 6.66–6.87), which was significantly longer than in patients who did not receive PCE (5.7 days; 95% confidence interval, 5.64–5.69). When looking at each stroke subtype separately, this association was evident for patients with ischemic stroke (7.4 versus 6.2 days). Conversely, PCE was associated with shorter LOS in patients with ICH (5.0 versus 8.3 days) or SAH (6 versus 12 days; Table 3).

**Table 3.**
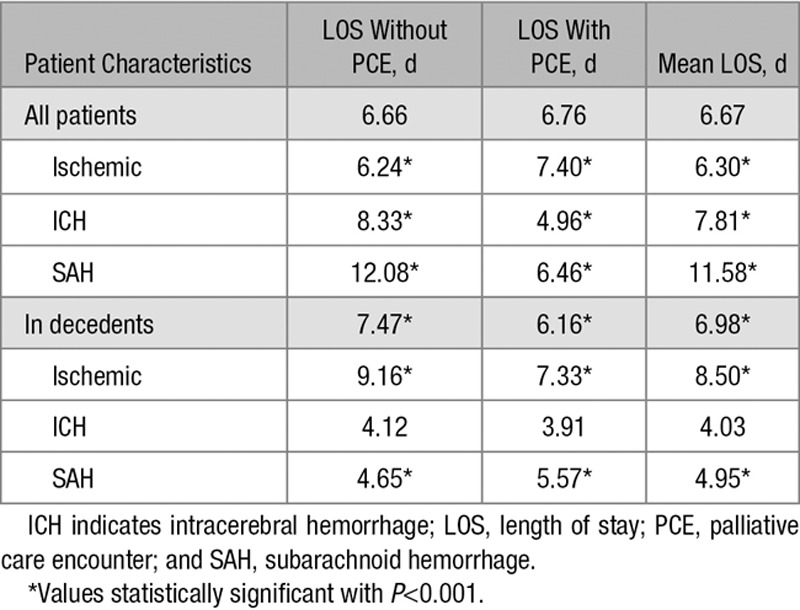
LOS With and Without PCE

### Palliative Care and Hospital Characteristics

**Figure 1.**
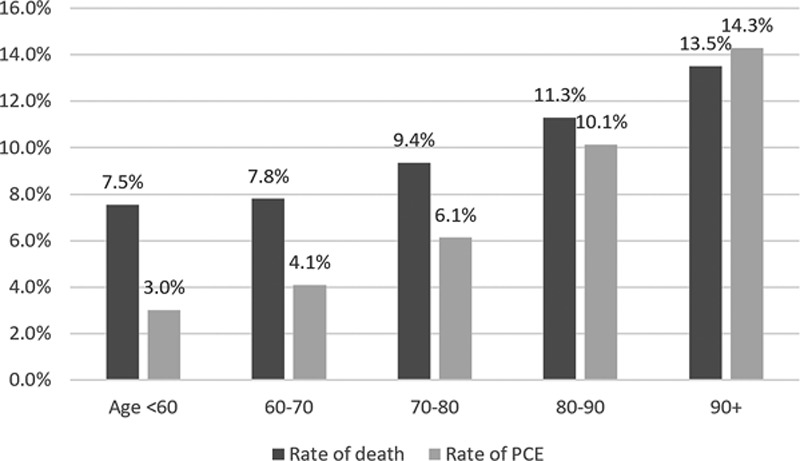
Rates of death and palliative care encounter (PCE) among patients with stroke by age.

Hospitals with higher PCE use included large hospitals (OR, 1.24 compared with small hospitals), urban teaching (OR, 1.1 compared with rural), nonprofit hospitals (OR, 1.22 compared with government hospitals), and western states (OR, 1.5 compared with northeast hospitals). In general, hospitals with higher mortality also had higher use of PCE. However, this trend comes with a wide variability showing some hospitals with low PCE use and high mortality, as well hospitals with low mortality and high PCE use (Figure 1).

### Palliative Care and Inpatient Mortality

Among all patients with stroke, 36 397 (9.2%) died in hospital, and the rate of death declined from 2010 (10.9%) to 2012 (9.8%; *P*<0.001; Table I in the online-only Data Supplement). Patient characteristics that were independently associated with higher mortality after stroke included older age (≥80; OR, 2.64 compared with <60), female sex (OR, 1.04), white race (OR, 0.77 for black versus white), ICH (OR, 4.76 compared with ischemic stroke), and non-Medicare insurance (OR self-pay 1.79 and private insurance 1.27 compared with Medicare). Hospital characteristics independently associated with higher mortality after stroke included small hospitals, hospitals in the northeast region, hospitals in rural areas, and public hospitals.

**Figure 2.**
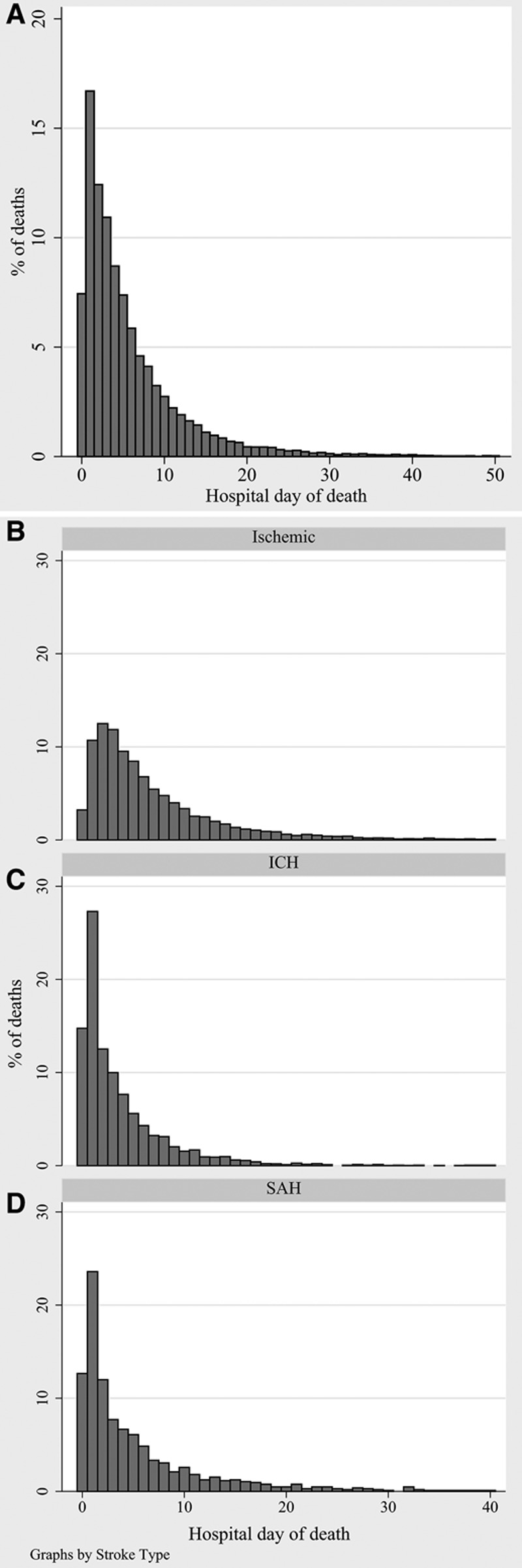
Proportion of deaths per hospital length of stay among patients who received palliative care encounter (**A**). All patients (**B** through **D**) by stroke type. ICH indicates intracerebral hemorrhage; and SAH, subarachnoid hemorrhage.

Among the patients who died, more than one third (38%) had received PCE (Table II in the online-only Data Supplement). The proportion of PCE was highest among patients dying with ICH (42%), followed by ischemic stroke (36%) and SAH (33%). Nonwhite races were less likely to die in hospital, and among those who died, nonwhites were significantly less likely to receive PCE. The rates of death and PCE both increased with age, whereas the difference between the 2 decreased: among young patients (<60 years), more than twice as many patients died than received PCE. In the oldest patients (>90), more patients received PCE than died (Figure 2).

**Figure 3.**
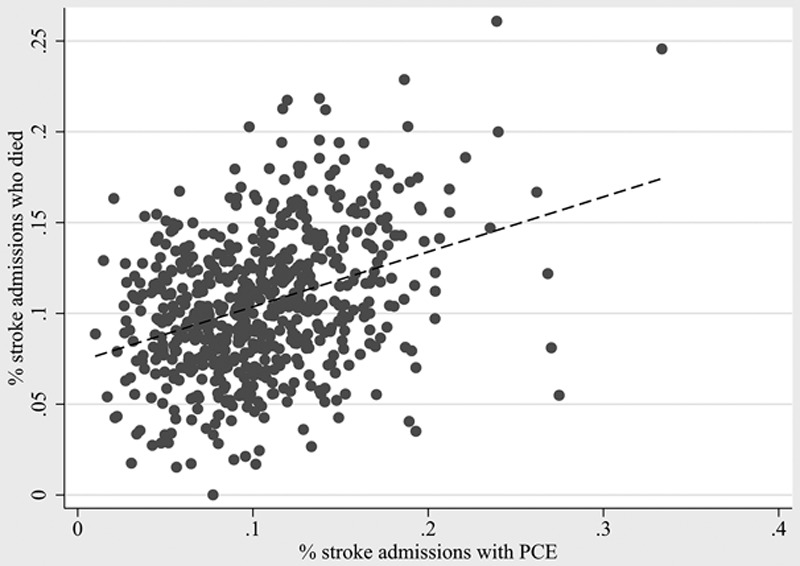
Hospital mortality and hospital palliative care encounter (PCE) use. Each dot represents one hospital. Hospitals with <10 stroke admissions or <10 palliative care encounters per year were excluded.

In the group of patients who died in hospital, the mean LOS was 7.0 days (95% confidence interval, 6.9–7.1). PCE use was associated with longer LOS in patients with ischemic stroke and with shorter LOS in patients with ICH and SAH. In decedents, PCE use was associated with a shorter LOS overall (6.2 versus 7.5 days) but with a longer LOS in patients with SAH (Table 3). In other words, PCE was associated with early death. The percentage of all PCE-related deaths was the highest in the earliest days of hospitalization both overall and for each stroke type (Figures 1 and 3).

## Discussion

Using a well-established database of inpatient admissions in the United States, we found an overall rate of coding of PCEs among patients with stroke of 6.2% and an inpatient mortality rate of 9.2%. We observed substantial variation across patient and hospital characteristics and a strong correlation between palliative care use and death. Our findings have important implications for the use of hospital mortality rates as a CMS quality measure.

### Palliative Care, Mortality, and Quality of Care Across Age, Race, and Sex

Consistent with the National Vital Statistics Report,^1^ we observed that stroke admissions and in-hospital mortality increased with age. We found that the rate of PCE increased with age, and this difference was especially pronounced among decedents, where less than half of those younger than 60 years of age received PCE but all of those older than 90 years(Figure 2). In other words, older patients who die do so in the setting of PCE, whereas younger patients are more likely to die without PCE. This finding suggests a lower emphasis on life-prolonging care among older patients with stroke but may not indicate poor quality care. On the contrary, a recent study showed that the presence of DNR orders in patients with acute ischemic stroke, highly associated with older age and mortality, did not predict a lower incidence of stroke quality of care indicators.^25^

Use of PCE and hospital mortality in the setting of PCE were also all more common in white people compared with blacks and Hispanics. This finding is consistent with a well-described racial variation in end-of-life care showing consistently lower rates of advance care planning,^26^ DNR orders,^27^ palliative care use^28^ and end-of-life discussions,^29^ and a higher rate of life-prolonging treatment, including PEG tube placement^30^ among black patients with serious illness. Quality care indicators, however, are observed less frequently in black patients with stroke compared with white patients,^31^ and hospital deaths occur alongside high adherence to high-quality, evidence-based stroke care.^16^

Although not as clear, evidence for the association of female sex with palliative care use, as suggested in our study, has some support in the literature. Previous studies in patients with stroke have indicated higher rates of DNR orders and withdrawal of life-sustaining treatments in women and whites^32–35^ but slightly lower quality of care.^36,37^

Finally, our results suggest substantial practice variation of PCE use, consistent with the variation previously shown in end-of-life care after stroke, in particular in regards to the use of DNR orders,^16,38^ and prognostication.^39^ The variation seen in our study may affect mortality in this group of patients, casting a shadow over the meaning and validity of mortality-based hospital comparisons that fail to account for PCE.

### PCE (V66.7) Versus Palliative Care Services

The report card published by the center to advance palliative care showed that access to palliative care specialist services in US hospitals has increased in the past decade, but that a variability persists in regards to hospital size, location, and tax status.^12^ In 2015, one third of US hospitals with 50 or more beds reported no palliative care services.^12^ The reports’ findings of a reduced rate of palliative care specialist services in smaller and nonacademic centers parallels the lower PCE rates in smaller, for-profit hospitals seen in our study, suggesting similar practice variations for specialist palliative care and palliative end-of-life care. Similarly, the geographic variation in palliative care specialist availability^12^ corresponds with our finding of a higher PCE rate in the western states of the United States without substantial variation in mortality rates.

### PCE and LOS

Among decedents, PCE was associated with a shorter LOS suggesting an earlier death through PCE and less days of aggressive life-sustaining treatment. LOS with PCE was longer in patients with ischemic stroke and shorter for ICH, which may be explained by a larger proportion of less-severe strokes on the one hand and later palliative care engagement for severe but nondeadly ischemic strokes on the other. This hypothesis was supported when we restricted the analysis to patients with ischemic stroke who died in the hospital: the trend reversed to shorter LOS with PCE. When looking only at patients with ICH, the association of PCE with shorter LOS was both seen in all patients with ICH and in decedents, possibly because of the high mortality and prognostic pessimism^40^ in this stroke type. For the small group of patients with SAH, PCE was associated with shorter LOS, but here, the trend reversed when we looked only at patients who died. One possible explanation may be a difference in the culture of the medical services, given that SAH is typically managed by different medical teams than ischemic stroke and ICH.

### Limitations

This study has several limitations, including those related to the retrospective analysis of the NIS database and the nature of an analysis based on ICD-9 coding. Large numbers in this data set lead to statistical significance even with small clinical changes. Owing to the nature of a preexisting database, important patient characteristics, such as stroke severity scales, are unavailable (eg, National Institutes of Health Stroke Scale). Second, ICD-9 coding is typically performed by the billing departments of hospitals based on language used by providers in their documentation. Provider documentation and billing guidelines may vary across individual departments, hospital types, geographic regions, and by individual administrative personnel. In addition, because the V66.7 code is not linked to reimbursement, the documentation may be less reliable. It is possible that our observations indicate an increase in the coding of palliative care rather than an increase in the actual use of palliative care over time. However, the patterns observed in this study correlate with other studies, suggesting a proportionate use of the PCE code. For example, the variability of PCEs across sampling year, age, region and hospital size, and ownership correlate with the availability of palliative care specialist services shown in the center to advance palliative care report card.^13^ Finally, documentation of PCE does not reflect the entirety of palliative care that a patient receives through primary or specialty palliative care. It also does not act as a surrogate for the degree to which goals of care, early comfort care measures, or surrogate decision making were addressed. Such services may be provided by the primary treating team without specific coding. More research is needed to build palliative and patient-centered care as a measurable healthcare quality metric.

### Conclusions

Palliative care is increasing among patients with stroke, especially in larger hospitals. Disparities and variability in PCE and mortality across age, sex, race, region, and hospital characteristics are apparent. When evaluating 30-day mortality as a marker of quality of care, the presence or absence of PCE needs to be taken into account.

## Disclosures

Dr Creutzfeldt receives support from the Cambia Health Foundation. The other authors report no conflicts.

## Supplementary Material
