# Supplementary material for: Palliative Care for Hospitalized Patients With Stroke: Results From the 2010 to 2012 National Inpatient Sample
Source: Stroke. 2017 Aug 17;48(9):2534–40. doi: 10.1161/STROKEAHA.117.016893 (PMC5571885; doi:10.1161/STROKEAHA.117.016893)
Supplement: Supplementary file 1 [file str-48-2534-s001.pdf]

ONLINE SUPPLEMENT

Palliative care for hospitalized stroke patients: results from the 2010-2012 National Inpatient Sample

Supplemental Tables

TABLE I: Trends in Palliative care utilization.

**Year**	**2010**	**2011**	**2012**
**Number of hospitals**	1001	990	3829
**Total number of stroke admissions**	130,418	137,846	127,230
**In-hospital deaths (% of stroke admissions)**	12,773 (9.8%)	12,314 (8.9%)	11,310 (8.9%)
**Total number of admissions receiving PCE**	7,061	8,810	8,773
**In-hospital stroke deaths receiving PCE (%)**	32.7%	37.9%	42.0%
**PCE per stroke admission (%)**	5.4%	6.3%	6.9%

Table II: Discharge characteristics. All $p < 0.01$.

Discharge characteristics		No Palliative Care<br>n (%)	Palliative Care<br>n (%)
**Discharge Status (all patients, 2010-2012)**			
	Alive at Discharge	347,782 (93.87)	10,933 (44.39)
	Died in Hospital	22,700 (6.13)	13,697 (55.61)
**Discharge Status (only patients with discharge status, 2010-2011)**			
	Alive at Discharge except hospice	199,955 (90.05)	1,932 (14.16)
	Died in Hospital	14,436 (6.50)	7,799 (57.20)
	Discharged to hospice	7,648 (3.45)	3,904 (28.64)
**Discharge Disposition (2010-2011)**			
	Routine	72,254 (32.54)	246 (1.80)
	Skilled Nursing Facility (SNF)	44,218 (19.91)	1,198 (8.79)
	Home Health Care	25,283 (11.39)	113 (0.83)
	Rehab facility	39,462 (17.77)	79 (0.58)
	Short-term hospital	6,631 (2.99)	69 (0.51)
	Expired in hospital	14,376 (6.47)	7,666 (56.22)
	Died in a medical facility	90 (0.04)	133 (0.09)
	Hospice--Home	2,287 (1.03)	1,132 (8.30)
	Hospice--Medical	5,361 (2.41)	2,772 (20.33)
	Others	12,077 (5.45)	348 (2.55)
